# Global Prevalence and Subtype Distribution of *Blastocystis* sp. in Rodent Populations: A Systematic Review and Meta‐Analysis

**DOI:** 10.1002/vms3.70178

**Published:** 2024-12-30

**Authors:** Amir Farzam, Amin Karampour, Seyedeh‐Sara Nazem‐Sadati, Parisa Sadat‐Madani, Ali Asghari

**Affiliations:** ^1^ Cellular and Molecular Research Center, Research Institute for Prevention of Non‐Communicable Diseases Qazvin University of Medical Sciences Qazvin Iran; ^2^ Non‐Communicable Diseases Research Center, Research Institute for Prevention of Non‐Communicable Diseases Qazvin University of Medical Sciences Qazvin Iran; ^3^ Medical Microbiology Research Center Qazvin University of Medical Sciences Qazvin Iran; ^4^ Health Products Safety Research Center Qazvin University of Medical Sciences Qazvin Iran

**Keywords:** *Blastocystis* sp, epidemiology, meta‐analysis, rodents, subtypes, systematic review

## Abstract

**Background:**

The present systematic review and meta‐analysis aimed to gather and analyse global data on the prevalence, subtypes (STs) distribution and zoonotic potential of *Blastocystis* sp. in rodents.

**Methods:**

A systematic literature search was performed across multiple databases (PubMed, Scopus, Web of Science and ProQuest) for studies published by 23 July 2024. The analysis included 34 studies/78 datasets, comprising 5661 samples from various rodent species across 15 countries. Statistical analyses were performed using comprehensive meta‐analysis (CMA) software, employing a random‐effects model to estimate pooled prevalence and 95% confidence intervals (CIs) and the *I* ^2^ index for assessing heterogeneity.

**Results:**

This review found that 16% (95% CI: 12.6%–20.2%) of rodents worldwide were infected with *Blastocystis* sp. Voles and squirrels exhibited the highest infection rates at 29.8% (95% CI: 14.7%–51%) and 28.8% (95% CI: 14.4%–49.2%), whereas civets and porcupines had the lowest rates at 9.5% (95% CI: 6.6%–13.6%) and 7.1% (95% CI: 3.3%–14.7%), respectively. The findings indicated that rodents can host various *Blastocystis* sp. STs (ST1–ST8, ST10, ST13, ST15, ST17), with several (ST1–ST8 and ST10) having zoonotic potential. Globally, ST4, ST5, ST1 and ST3 were the most commonly reported STs in rodents. China and the UK showed the highest ST diversity in rodents, with 10 (ST1‐ST7, ST10, ST13, ST17) and 7 (ST1‐ST5, ST10, ST15) distinct STs, respectively. ST6, ST7 and ST13 were unique to China, whereas ST15 was found only in the United Kingdom. Squirrels, rats, mice and voles had the highest ST diversity of *Blastocystis* sp., with 8, 7, 5 and 5 distinct STs, respectively. Notably, ST6 and ST13 were unique to squirrels, ST7 only appeared in rats, and ST15 was found only in voles. Most ST1, ST3–ST5 and ST17 came from Asia. ST6, ST7 and ST13 were also isolated there, whereas ST15 was only found in Europe. ST17 was reported in Africa, ST4 and ST17 in North America, and ST1–ST3 and ST8 in South America.

**Conclusions:**

This review emphasizes the widespread presence of *Blastocystis* sp. in rodent populations globally, underscoring the need for continued surveillance and research into its zoonotic potential.

## Graphical abstract

Overall, 16% of rodents worldwide were infected with *Blastocystis* sp. (95% CI: 12.6%–20.2%). Rodents host various *Blastocystis* sp. subtypes (STs), including ST1‐ST8, ST10, ST13, ST15 and ST17. The most commonly reported STs in rodents globally were ST4, ST5, ST1 and ST3.

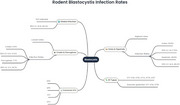

## 1 Introduction

*Blastocystis* sp. is an anaerobic eukaryotic protist, the only stramenopile known to infect humans (Stensvold and Clark 2016). Over 1 billion people are infected globally, making it likely the most common intestinal parasite (Scanlan and Stensvold 2013). This protist is primarily transmitted through the faecal–oral route, either by direct contact with infected hosts or by consuming contaminated food or water (Tan 2004).

The presence of *Blastocystis* sp. in various pets, livestock and wildlife indicates that these reservoir species play a key role in transmitting the parasite to humans and vice versa (Hublin, Maloney, and Santin 2021). *Blastocystis* sp. displays significant genetic diversity, with 44 recognized subtypes (STs) (Koehler et al. 2024; Šejnohová et al. 2024). Of these, only 16 are zoonotic (ST1–ST10, ST12, ST14, ST16, ST23, ST35 and ST41) and have been found in humans, with ST1–ST4 representing over 95% of reported cases (Maloney et al. 2022; Hernández et al. 2023).

Previous findings revealed that certain *Blastocystis* sp. STs, such as ST4, positively affect intestinal commensal bacteria and inhibit pathogenic growth, indicating a potential role in gut health. Additionally, *Blastocystis* sp. colonization may beneficially alter the gut bacterial composition, enhance short‐chain fatty acid (SCFA) production, and influence immune responses (Th2 and Treg) in various murine models. ST1, another common human subtype, also demonstrated positive effects on gut microbiota and adaptive immune responses, further supporting the notion that some *Blastocystis* sp. STs may promote health. Although evidence supports that *Blastocystis* sp. is primarily a commensal organism, exceptions exist, notably ST7, which exhibits pathogenic potential. Both in vitro and rodent studies indicated that ST4 can cause mild inflammation, highlighting caution in universally classifying *Blastocystis* sp. as commensal, given the genetic diversity among STs. Future research should explore the mechanisms of *Blastocystis* sp. interactions with gut microbiota and their effects on microbial‐derived metabolites like SCFAs and bile acids. Additionally, further studies in humans or naturally colonized animals are needed to clarify the role of *Blastocystis* sp. in immunity and gut health (Deng et al. 2021; Deng and Tan 2022; Deng et al. 2022, 2023).

Rodents play a crucial role in every terrestrial ecosystem. With nearly 36 families and 2552 species, they are the most diverse and representative group, comprising 40% of all mammals (Islam 2021). Outbreaks of rodent‐borne zoonotic diseases, such as bubonic plague, leishmaniasis and typhus, have occurred worldwide (Rabiee et al. 2018; Ganjeer et al. 2021). Moreover, some animals, such as cats and dogs, can consume rodents, facilitating human infections and transmission of parasites like *Blastocystis* sp. between species (Morelli et al. 2021; Macpherson et al. 2022). These cases underscore the urgent need for a comprehensive framework to study infections like *Blastocystis* sp. in rodent populations. Hence, this review aimed to statistically analyse and summarize the prevalence and ST distribution of *Blastocystis* sp. in rodents, given the potential pathogenicity and zoonotic nature of some *Blastocystis* sp. STs.

## 2 Materials and Methods

### 2.1 Ethical Approval

The present study with the code IR.QUMS.REC.1403.231 was approved by the ethics committee of Qazvin University of Medical Sciences, Qazvin, Iran.

### 2.2 Type and Design of the Study

The current study was a systematic review and meta‐analysis conducted during 2024. The studies were reported according to the PRISMA (Preferred Reporting Items for Systematic Reviews and Meta‐Analysis) checklist guidelines (Moher et al. 2015).

### 2.3 Search Strategy

Four key global databases (Medline/PubMed, ProQuest, Scopus and the Web of Knowledge) were searched for articles published up to 23 July 2024. Google Scholar and the references of included articles were also probed to identify any overlooked studies. The search utilized Medical Subject Headings (MeSH) terms alone or in combination: (‘Intestinal Parasites’ OR ‘Parasitic Infections’ OR ‘*Blastocystis* sp.’) AND (‘Prevalence’ OR ‘Epidemiology’ OR ‘Frequency’ OR ‘Occurrence’) AND (‘Subtype’ OR ‘Subtyping’) AND (Rodents). The collected data were imported into Endnote X9, where duplicates were automatically removed, and the remaining studies were evaluated by two researchers on the basis of their titles and abstracts.

### 2.4 Inclusion and Exclusion Criteria

This study included all published descriptive cross‐sectional studies with accessible full texts on the prevalence and distribution of *Blastocystis* sp. STs in rodents, without restrictions on time, language, or geography. Excluded were review studies, case studies, commentaries, human studies, studies involving animals other than rodents, experimental studies, studies/datasets conducted based on one sample and those with unclear or ambiguous results and sample sizes.

### 2.5 Quality Evaluation Checklist

In addition to the inclusion and exclusion criteria, the studies were evaluated using the Joanna Briggs Institute (JBI) checklist (Munn et al. 2014), which consists of nine questions/parts regarding sample size, statistical analysis, sample explanations, confounding factors and subgroup analysis. Each study was rated with a yes/no response. Studies scoring 4–6 and ≥7 were classified as medium and high quality, respectively, whereas those scoring ≤3 were excluded from the review.

### 2.6 Meta‐Analysis

Statistical analyses were conducted using comprehensive meta‐analysis (CMA) v3 software, with *p* values below 0.05 considered statistically significant (Mahdavi et al. 2024). A random‐effects model was used to assess the prevalence of *Blastocystis* sp. in rodents, providing pooled prevalence and 95% confidence intervals (CIs). A subgroup analysis assessed the weighted prevalence of rodent infections by WHO regions, countries, publication years, continents and sample sizes. A forest plot illustrated the pooled prevalence and 95% CIs, whereas a funnel plot evaluated publication bias. Heterogeneity among studies was assessed using the *I* ^2^ index, with values categorized as low (below 25%), moderate (25%–50%) and high (above 50%). Additionally, a sensitivity analysis was performed to evaluate the impact of excluding specific studies on the final weighted prevalence of *Blastocystis* sp.

## 3 Results

### 3.1 Selection of Studies

**FIGURE 1:**
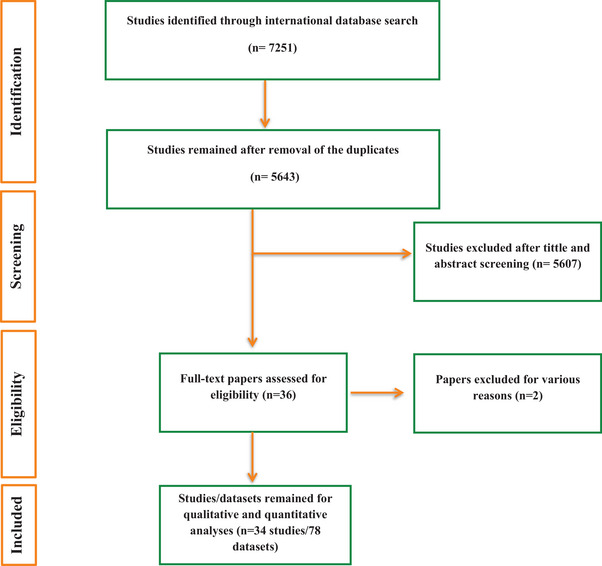
Flowchart depicting the process of included studies in the present review.

Expert researchers thoroughly searched four international databases, identifying 7251 initial records. After removing duplicates and reviewing the 5643 remaining studies, they ultimately selected 36 articles. Finally, a quality assessment using JBI criteria excluded two additional studies, resulting in 34 relevant studies (78 datasets) (Alfellani et al. 2013; Ramírez et al. 2014; Seifollahi et al. 2016; Yoshikawa et al. 2016; Cian et al. 2017; Mohaghegh et al. 2018; Wang et al. 2018, 2024; Betts et al. 2018, Betts, Gentekaki, and Tsaousis 2020; AbuOdeh et al. 2019; Valenca‐Barbosa et al. 2019; Xiao et al. 2019; Li et al. 2020; Martinez‐Hernandez et al. 2020; Mohammadpour et al. 2020; Oliveira‐Arbex et al. 2020; Li et al. 2020; Chai et al. 2020; Liu et al. 2021, 2022, 2024; Malatyalı et al. 2021; Masuda et al. 2021; Rudzińska et al. 2021; Song et al. 2021; Chen et al. 2021; Deng et al. 2021; Tantrawatpan et al. 2023; Zhao et al. 2023; Martínez‐Hernández et al. 2024; Bastaminejad et al. 2024; Shan et al. 2024; Gao et al. 2024) meeting the inclusion criteria for this study (Figure 1). Of note, the search, conducted without an initial time limit, continued until 23 July 2024, including any articles that met the study criteria. The included studies began in 2013 (Table 1), indicating that no relevant studies existed before that year.

**TABLE 1:** Key information from 34 articles/78 datasets on the occurrence and subtypes (ST) distribution of *Blastocystis* sp. in rodents.

Author (year)	Host (scientific name)	Host species	Time tested	Country	Total no.	Infected no.	Prevalence (%)	Method	STs^d^
Alfellani et al. (2013)	Bank vole (*Clethrionomys glareolus*)	Voles	UC^a^	Poland	6	0	0	MOL^b^	—
Alfellani et al. (2013)	Bank vole (*Clethrionomys glareolus*)	Voles	UC	The United Kingdom	32	1	3.1	MOL	ST5 (1)
Alfellani et al. (2013)	Chinchilla (*Chinchilla lanigera*)	Chinchillas	UC	Belgium	5	2	40	MOL	ST3 (2)
Alfellani et al. (2013)	Wood mouse (*Apodemus sylvaticus*)	Mice	UC	The United Kingdom	13	1	7.7	MOL	ST3 (1)
Alfellani et al. (2013)	Gundi (*Ctenodactylus gundi*)	Gundis	UC	Libya	4	1	25	MOL	ST17 (1)
Ramírez et al. (2014)	Black rat (*Rattus rattus*)	Rats	UC	Colombia	10	3	30	MOL	ST2 (3)
Seifollahi et al. (2016)	Rodent spp.	Rodent spp.	UC	Iran	52	10	19.2	^MIC^ ^c^	ND (10)
Yoshikawa et al. (2016)	Rodent spp.	Rodent spp.	2010–2012	Indonesia	77	10	13	MOL	ST4 (9), ND^e^ (1)
Cian et al. (2017)	Brown rat (*Rattus norvegicus*)	Rats	2014–2015	France	2	1	50	MOL	ST4 (1)
Cian et al. (2017)	Capybara (*Hydrochoerys hydrochaeris*)	Capybaras	2014–2015	France	5	3	60	MOL	ST2 (1), ST5 (1), ND (1)
Cian et al. (2017)	House mouse (*Mus musculus*)	Mice	2014–2015	France	2	0	0	MOL	—
Cian et al. (2017)	Indian crested porcupine (*Hystrix indica*)	Porcupines	2014–2015	France	2	0	0	MOL	—
Cian et al. (2017)	Patagonian mara (*Dolichotis patagonum*)	Patagonian mara	2014–2015	France	3	0	0	MOL	—
Betts et al. (2018)	Red squirrel (*Sciurus vulgaris*)	Squirrels	2016–2017	The United Kingdom	3	2	66.7	MOL	ST4 (1), ND (1)
Betts et al. (2018)	Water Vole (*Arvicola amphibius*)	Voles	2016–2017	The United Kingdom	11	10	90.9	MOL	ST1 (1), ST4 (36), ST10 (1)
Mohaghegh et al. (2018)	Wistar rat	Rats	UC	Iran	60	33	55	MIC	ND (33)
Wang et al. (2018)	Brown rat (*Rattus norvegicus*)	Rats	2015–2017	China	108	4	3.7	MOL	ST4 (4)
Xiao et al. (2019)	Flying squirrel (*Trogopterus xanthipes*)	Squirrels	2017	China	69	28	30.4	MOL	ST1 (12), ST3 (7), ST13 (9)
AbuOdeh et al. (2019)	Squirrel	Squirrels	UC	The UAE	6	3	50	MOL	ST4 (2), ST17 (1)
Valenca‐Barbosa et al. (2019)	Rodent spp.	Rodent spp.	UC	Brazil	11	7	63.6	MOL	ST3 (1), ST8 (1), ND (5)
Oliveira‐Arbex et al. (2020)	Capybara (*Hydrochoerus hydrochaeris*)	Capybaras	UC	Brazil	23	2	8.7	MOL	ST1, ST8
Chai et al. (2020)	Eurasian red squirrel (*Sciurus vulgaris*)	Squirrels	2018–2019	China	72	7	9.7	MOL	ST4 (7)
Chai et al. (2020)	Eastern chipmunk (*Tamias striatus*)	Squirrels	2018–2019	China	171	8	4.7	MOL	ST4 (8)
Chai et al. (2020)	Chinchilla (*Chinchilla lanigera*)	Chinchillas	2018–2019	China	72	3	4.2	MOL	ST4 (2), ST17 (1)
Chai et al. (2020)	Guinea pig (*Cavia porcellus*)	Guinea pigs	2018–2019	China	90	12	13.3	MOL	ST4 (12)
Chai et al. (2020)	Chinese striped hamster (*Cricetulus barabensis*)	Mice	2018–2019	China	98	12	12.2	MOL	ST4 (12)
Li et al. (2020)	Sprague–Dawley rat	Rats	2019	China	151	17	11.3	MOL	ST4 (16), ST7 (1)
Li et al. (2020)	Wistar rat	Rats	2019	China	104	9	8.7	MOL	ST4 (9)
Li et al. (2020)	Spontaneous hypertensive rat	Rats	2019	China	100	3	3	MOL	ST4 (1), ST7 (2)
Li et al. (2020)	Patagonian mara (*Dolichotis patagonum*)	Patagonian mara	UC	China	15	3	20	MOL	ST4 (3)
Martinez‐Hernandez et al. (2020)	Kangaroo rat (*Heteromyidae sp*.)	Rats	2015–2016	Mexico	8	4	50	MOL	ST4 (1), ST17 (2), ND (1)
Martinez‐Hernandez et al. (2020)	*Rodent* spp.	*Rodent* spp.	2015–2016	Mexico	22	0	0	MOL	—
Betts, Gentekaki, and Tsaousis (2020)	Red squirrel (*Sciurus vulgaris*)	Squirrels	2016–2019	The United Kingdom	5	3	60	MOL	ST2 (3), ST4 (1)
Betts, Gentekaki, and Tsaousis (2020)	Water vole (*Arvicola amphibius*)	Voles	2016–2019	The United Kingdom	74	27	36.5	MOL	ST1 (3), ST4 (78), ST10 (1), ST15 (3), ND (2)
Mohammadpour et al. (2020)	Brown rat (*Rattus norvegicus*)	Rats	2016–2018	Iran	127	20	15.8	MOL	ST1 (4), ST3 (4), ST4 (12)
Rudzińska et al. (2021)	Capybara (*Hydrochoerus hydrochaeris*)	Capybaras	2018–2019	Poland	6	0	0	MOL	—
Liu et al. (2021)	Pallas's squirrel (*Callosciurus erythraeus*)	Squirrels	2018	China	171	10	5.8	MOL	ST1 (1), ST3 (1), ST5 (4), ST6 (4)
Masuda et al. (2021)	Pallas's squirrel (*Callosciurus erythraeus*)	Squirrels	UC	Japan	423	186	44	MOL	ST4 (85), ND (101)
Chen et al. (2021)	Porcupine (*Hystrix hodgsoni*)	Porcupines	2020	China	7	1	14.3	MOL	ST1 (1)
Song et al. (2021)	Bamboo rat (*Rhizomys sinensis*)	Rats	UC	China	480	22	4.6	MOL	ST4 (17), ST5 (5)
Malatyalı et al. (2021)	Laboratory rat (*Sprague–Dawley rats*)	Rats	UC	Turkey	54	33	61.1	MOL	ST4 (33)
Deng et al. (2021)	*Rodent* spp.	Rodent spp.	2017–2019	China	33	6	18.2	MOL	ST4 (2), ST17 (4)
Liu et al. (2022)	Coypu (*Myocastor coypus*)	Coypus	2018–2019	China	308	44	14.3	MOL	ST4 (33), ST5 (3), ND (8)
Tantrawatpan et al. (2023)	Capybara (*Hydrochoerus hydrochaeris*)	Capybaras	UC	Thailand	3	0	0	MOL	—
Tantrawatpan et al. (2023)	Guinea pig (*Cavia porcellus Anas*)	Guinea pigs	UC	Thailand	12	0	0	MOL	—
Tantrawatpan et al. (2023)	Malayan porcupine (*Hystrix brachyura*)	Porcupines	UC	Thailand	5	1	20	MOL	ST8 (1)
Tantrawatpan et al. (2023)	Rice field rat (*Rattus argentiventer*)	Rats	UC	Thailand	10	1	10	MOL	ST4 (1)
Tantrawatpan et al. (2023)	Pllas's squirrel (*Callosciurus erythraeus*)	Squirrels	UC	Thailand	10	6	60	MOL	ST4 (4), ND (2)
Tantrawatpan et al. (2023)	Variable squirrel (*Callosciurus finlaysonii*)	Squirrels	UC	Thailand	2	1	50	MOL	ST4 (1)
Zhao et al. (2023)	Asiatic brush‐tailed porcupine (*Atherurus macrourus*)	Porcupines	2017–2021	China	257	12	4.7	MOL	ST4(11), ND (1)
Zhao et al. (2023)	Bamboo rat (*Rhizomys pruinosus*)	Rats	2017–2021	China	360	8	2.2	MOL	ST4 (8)
Zhao et al. (2023)	Masked palm civet (*Paguma larvata*)	Civets	2017–2021	China	283	27	9.5	MOL	ST1 (1), ST5 (26)
Liu et al. (2024)	Brown rat (*Rattus norvegicus*)	Rats	2023–2024	China	195	63	32.3	MOL	ST1 (4), ST2 (2), ST4 (57)
Liu et al. (2024)	House mouse (*Mus musculus*)	Mice	2023–2024	China	106	16	15.1	MOL	ST4 (16)
Liu et al. (2024)	Striped field mouse (*Apodemus agrarius*)	Mice	2023–2024	China	89	18	20.2	MOL	ST4 (17), ST10 (1)
Liu et al. (2024)	Striped hamster (*Cricetulus barabensis*)	Mice	2023–2024	China	96	36	37.5	MOL	ST3 (4), ST4 (32)
Shan et al. (2024)	Asian house rat (*Rattus tanezumi*)	Rats	2021–2023	China	136	41	30.1	MOL	ST1 (2), ST3 (2), ST4 (8), ST5 (29)
Shan et al. (2024)	Brown rat (*Rattus norvegicus*)	Rats	2021–2023	China	58	42	72.4	MOL	ST1 (1), ST2 (1), ST4 (35), ST5 (5)
Shan et al. (2024)	House mouse (*Mus musculus*)	Mice	2021–2023	China	25	3	12	MOL	ST3 (1), ST4 (1), ST5 (1)
Martínez‐Hernández et al. (2024)	Brown rat (*Rattus norvegicus*)	Rats	2020	Mexico	52	17	32.7	MOL	ND (17)
Martínez‐Hernández et al. (2024)	House mouse (*Mus musculus*)	Mice	2020	Mexico	33	4	12.1	MOL	ND (4)
Bastaminejad et al. (2024a)	House mouse (*Mus musculus*)	Mice	2020	Iran	40	2	5	MOL	ST1 (1), ND (1)
Bastaminejad et al. (2024a)	Brown rat (*Rattus norvegicus*)	Rats	2020	Iran	40	3	7.5	MOL	ST1 (1), ST4 (1), ND (1)
Bastaminejad et al. (2024a)	Black rat (*Rattus rattus*)	Rats	2020	Iran	40	3	7.5	MOL	ST4 (2), ND (1)
Wang et al. (2024)	Brown rat (*Rattus norvegicus*)	Rats	2023	China	155	4	2.6	MOL	ST4 (4)
Wang et al. (2024)	Asian rat (*Rattus tanezumi*)	Rats	2023	China	86	3	3.5	MOL	ST4 (3)
Wang et al. (2024)	Chinese white‐bellied rat (*Niviventer confucianus*)	Rats	2023	China	75	13	17.3	MOL	ST1 (1), ST4 (11), ST7 (1)
Wang et al. (2024)	Striped field mouse (*Apodemus Agrarius Pallas*)	Mice	2023	China	36	1	2.8	MOL	ST4 (1)
Wang et al. (2024)	Lesser ricefield rat (*Rattus losea*)	Rats	2023	China	18	1	5.6	MOL	ST4 (1)
Gao et al. (2024)	Buff‐breasted rat (*Rattus flavipectus*)	Rats	2023–2024	China	39	3	7.7	MOL	ST4 (2), ST5 (1)
Gao et al. (2024)	Reed vole (*Microtus fortis*)	Voles	2023–2024	China	139	39	28.1	MOL	ST4 (39)
Gao et al. (2024)	Brown rat (*Rattus norvegicus*)	Rats	2023–2024	China	31	2	6.4	MOL	ST4 (2)
Gao et al. (2024)	Striped field mouse (*Apodemus agrarius*)	Mice	2023–2024	China	14	1	7.1	MOL	ST4 (1)
Gao et al. (2024)	Greater bandicoot rat (*Bandicota indica*)	Rats	2023–2024	China	39	1	2.5	MOL	ST4 (1)
Gao et al. (2024)	*Rattus rattus sladeni*	Rats	2023–2024	China	5	2	40	MOL	ST4 (2)
Gao et al. (2024)	Lesser ricefield rat (*Rattus losea*)	Rats	2023–2024	China	41	6	14.6	MOL	ST4 (6)
Gao et al. (2024)	White‐bellied Rat (*Niviventer lotipes*)	Rats	2023–2024	China	23	0	0	MOL	—
Gao et al. (2024)	House mouse (*Mus musculus*)	Mice	2023–2024	China	13	0	0	MOL	—

^a^ Unclear.

^b^ Molecular detection.

^c^ Microscopic detection.

^d^ Subtypes.

^e^ Not determined.

^*^ The higher number of STs compared to positive cases in some studies is due to the presence of the mixed infections with multiple STs and the analysis of multiple samples from a rodent.

### 3.2 Details of Included Studies

This review included 34 studies/78 datasets, comprising 5661 samples from various rodent species across 15 countries. Our results were based on studies published from 2013 to 2024, with sample sizes ranging from 2 to 480. Belgium, Colombia, Indonesia, Japan, Libya, Turkey and the UAE had only one study/dataset on *Blastocystis* sp. in rodents, whereas other countries had more than one study/dataset. Of the included studies, only 2 utilized microscopic methods for diagnosing *Blastocystis* sp., whereas molecular methods accounted for 32 studies/76 datasets. Of the 34 studies, 20 studies/64 datasets reported the distribution of *Blastocystis* sp. STs (Table 1). The JBI checklist evaluation classified 11 studies as high quality (>6 points) and 23 articles as moderate quality (4–6 points) (Table S1).

### 3.3 Global Prevalence of *Blastocystis* sp. in Rodents

**FIGURE 2:**
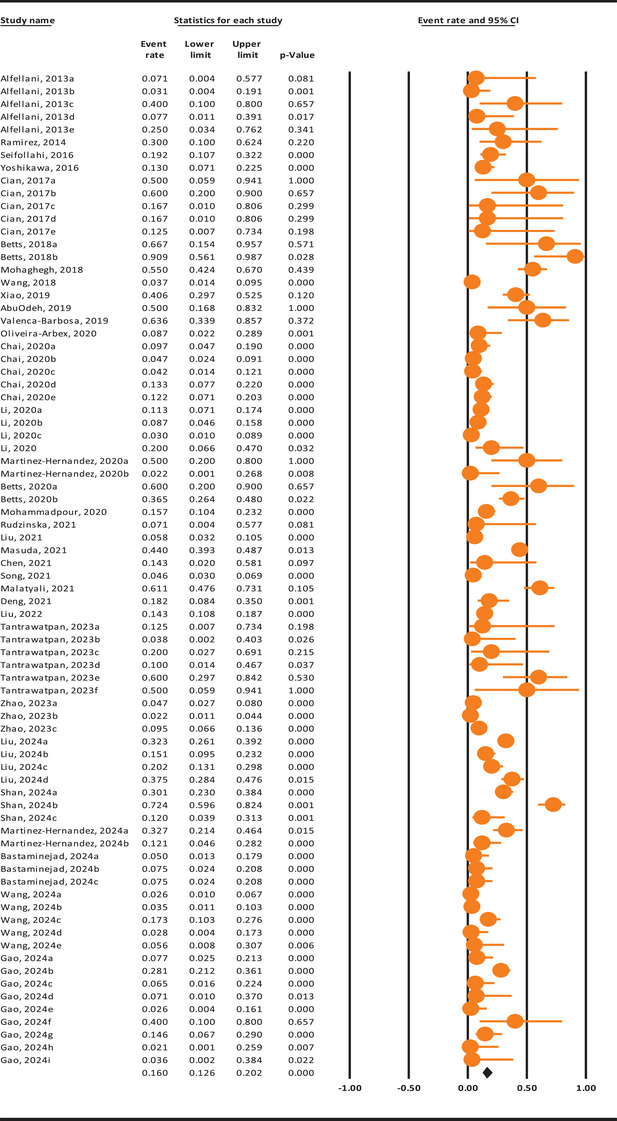
The pooled prevalence of *Blastocystis* sp. in rodents based on data from the included studies, using a random‐effects model and 95% confidence intervals. Orange colours indicate the event rate/prevalence reported in each study, whereas the black colour represents the final weighted prevalence.

The global prevalence of *Blastocystis* sp. in rodents was estimated at 16% (95% CI: 12.6%–20.2%) (Figure 2), exhibiting significant heterogeneity (*Q* = 750.1, *I* ^2^ = 89.7%, *p* < 0.001).

### 3.4 Worldwide Prevalence of *Blastocystis* sp. in Rodents Based On Species

**FIGURE 3:**
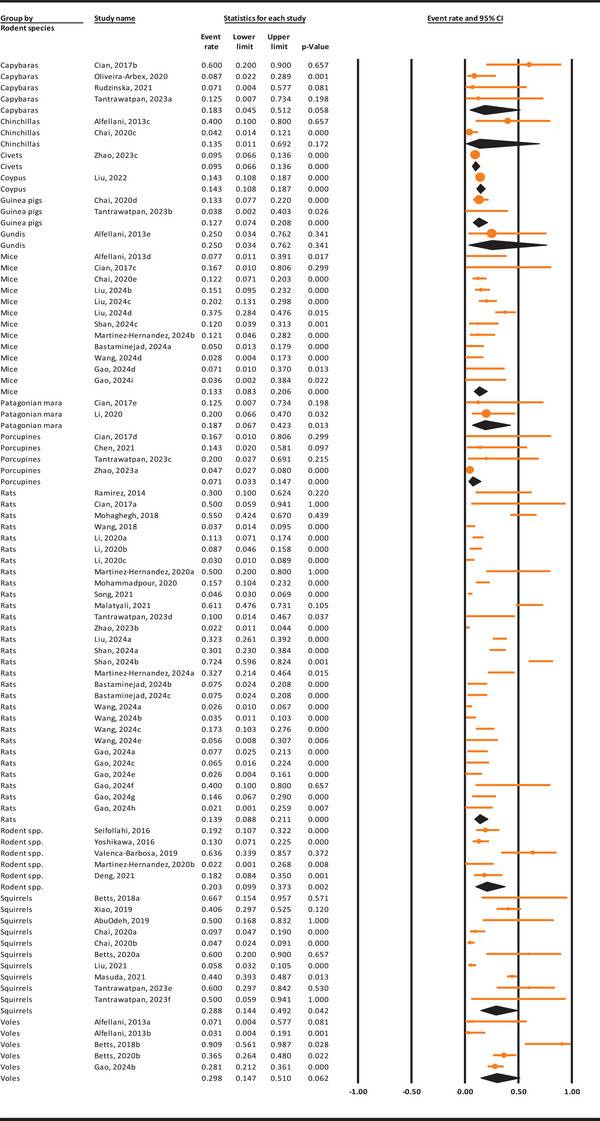
The pooled prevalence of *Blastocystis* sp. in rodents based on different species, using a random‐effects model and 95% confidence intervals.

The weighted prevalence of *Blastocystis* sp. in rodents based on species is shown in Figure 3. In brief, voles and squirrels exhibited the highest infection rates at 29.8% (95% CI: 14.7%–51%) and 28.8% (95% CI: 14.4%–49.2%), whereas civets and porcupines had the lowest rates at 9.5% (95% CI: 6.6%–13.6%) and 7.1% (95% CI: 3.3%–14.7%), respectively.

### 3.5 ST Distribution of *Blastocystis* sp. in Rodents

The findings indicated that rodents can host various *Blastocystis* sp. STs (ST1–ST8, ST10, ST13, ST15, ST17), with several (ST1–ST8 and ST10) having zoonotic potential (Table 1). Globally, ST4, ST5, ST1 and ST3 were the most commonly reported STs in rodents (Table 2).

**TABLE 2:** Subtypes (STs) distribution of *Blastocystis* sp. in rodents by countries and rodent species.

Variables	Dataset no.	Total samples (no.)	Infected samples (no.)	Reported STs (no.)												
				ST1	ST2	ST3	ST4	ST5	ST6	ST7	ST8	ST10	ST13	ST15	ST17	ND^b^
**Countries**																
Belgium	1	5	2	—	—	2	—	—	—	—	—	—	—	—	—	—
Brazil	2	34	9	1	—	1	—	—	—	—	2	—	—	—	—	5
China	40	4368	531	23	3	15	384	74	4	4	—	1	9	—	5	9
Colombia	1	10	3	—	3	—	—	—	—	—	—	—	—	—	—	—
France	5	14	4	—	1	—	1	1	—	—	—	—	—	—	—	1
Indonesia	1	77	10	—	—	—	9	—	—	—	—	—	—	—	—	1
Iran	6	359	71	6	—	4	15	—	—	—	—	—	—	—	—	46
Japan	1	423	186	—	—	—	85	—	—	—	—	—	—	—	—	101
Libya	1	4	1	—	—	—	—	—	—	—	—	—	—	—	1	—
Mexico	4	115	25	—	—	—	1	—	—	—	—	—	—	—	2	22
Poland	2	12	0	—	—	—	—	—	—	—	—	—	—	—	—	—
Thailand	6	42	9	—	—	—	6	—	—	—	1	—	—	—	—	2
Turkey	1	54	33	—	—	—	33	—	—	—	—	—	—	—	—	—
The UAE	1	6	3	—	—	—	2	—	—	—	—	—	—	—	1	—
The United Kingdom	6	138	44	4	3	1	116	1	—	—	—	2	—	3	—	3
**Total no. species**	**78**	**5661**	**931**	**34**	**10**	**23**	**652**	**76**	**4**	**4**	**3**	**3**	**9**	**3**	**9**	**190**
Voles	5	262	77	4	—	—	153	1	—	—	—	2	—	3	—	2
Chinchillas	2	77	5	—	—	2	2	—	—	—	—	—	—	—	1	—
Mice	12	565	94	1	—	6	80	1	—	—	—	1	—	—	—	5
Gundis	1	4	1	—	—	—	—	—	—	—	—	—	—	—	1	—
Rats	29	2607	362	13	6	6	238	40	—	4	—	—	—	—	2	53
Rodent spp.	5	195	33	—	—	1	11	—	—	—	1	—	—	—	4	16
Capybaras	4	37	5	1	1	—	—	1	—	—	1	—	—	—	—	1
Porcupines	4	271	14	1	—	—	11	—	—	—	1	—	—	—	—	1
Patagonian mara	2	18	3	—	—	—	3	—	—	—	—	—	—	—	—	—
Squirrels^a^	10	932	254	13	3	8	109	4	4	—	—	—	9	—	1	104
Guinea pigs	2	102	12	—	—	—	12	—	—	—	—	—	—	—	—	—
Coypus	1	308	44	—	—	—	33	3	—	—	—	—	—	—	—	8
Civets	1	283	27	1	—	—	—	26	—	—	—	—	—	—	—	—
**Total no**.	**78**	**5661**	**931**	**34**	**10**	**23**	**652**	**76**	**4**	**4**	**3**	**3**	**9**	**3**	**9**	**190**

^a^ A dataset related to chipmunk was included in this group.

^b^ Not determined.

^*^ The higher number of STs compared to positive cases in some studies is due to the presence of the mixed infections with multiple STs and the analysis of multiple samples from a rodent.

### 3.6 ST Distribution of *Blastocystis* sp. in Rodents by Countries, Rodent Species and Continents

**FIGURE 4:**
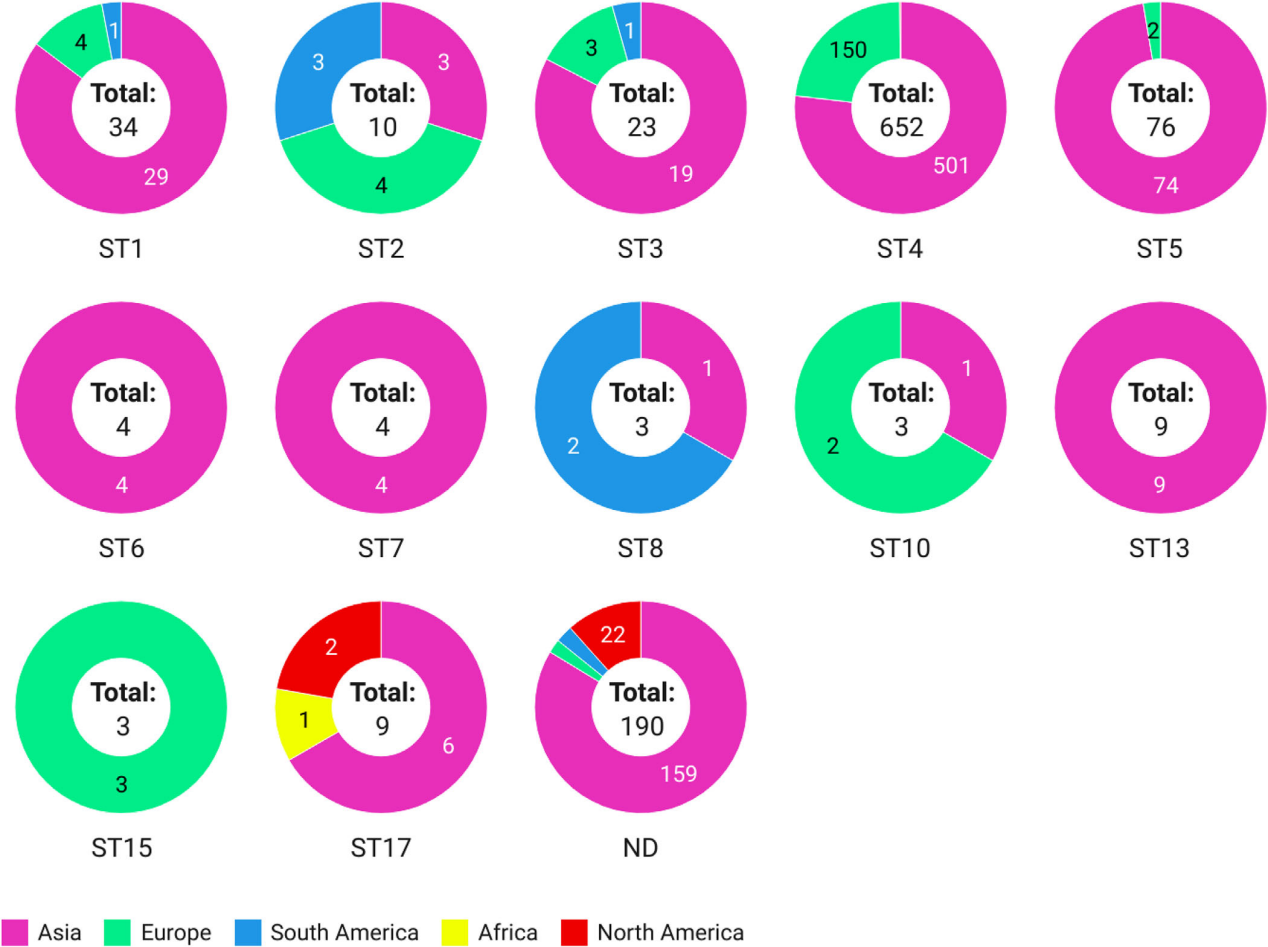
ST distribution of *Blastocystis* sp. in rodents by continents. STs, subtypes.

The distributions of *Blastocystis* sp. STs in rodents by countries, species and continents are summarized in Table 2 and Figure 4. In total, STs were reported in 14 countries. The highest numbers of samples were from China (40 datasets, 4368 samples), Japan (1 dataset, 423 samples), Iran (6 datasets, 359 samples), the United Kingdom (6 datasets, 138 samples) and Mexico (4 datasets, 115 samples). China and the United Kingdom exhibited the greatest ST diversity in rodents, with 10 (ST1–ST7, ST10, ST13, ST17) and 7 (ST1–ST5, ST10, ST15) distinct STs reported, respectively. Notably, ST6, ST7 and ST13 were only found in China, whereas ST15 was exclusively reported in the United Kingdom.

Species‐based analysis showed that the majority of samples were reported from rats (29 datasets, 2607 samples), squirrels (10 datasets, 932 samples), mice (12 datasets, 565 samples), coypus (1 dataset, 308 samples), civets (1 dataset, 283 samples), porcupines (4 datasets, 271 samples) and voles (5 datasets, 262 samples). Squirrels, rats, mice and voles displayed the highest ST diversity of *Blastocystis* sp., with 8 (ST1–ST6, ST13 and ST17), 7 (ST1–ST5, ST7 and ST17), 5 (ST1, ST3–ST5 and ST10) and 5 (ST1, ST4, ST5, ST10 and ST15) distinct STs, respectively. Interestingly, ST6 and ST13 were reported solely in squirrels, ST7 only in rats and ST15 only in voles.

Most of ST1, ST3–ST5 and ST17 were reported from Asia. ST6, ST7 and ST13 were isolated from Asia, whereas ST15 was reported only in Europe. ST17 was the only one reported from Africa, ST4 and ST17 from North America and ST1–ST3 and ST8 from South America.

### 3.7 Pooled Prevalence of *Blastocystis* sp. in Rodents Based On Subgroups

Table 3 and Figures S1–S5 show the prevalence of *Blastocystis* sp. in rodents by subgroup.

**TABLE 3:** Subgroup analysis of *Blastocystis* sp. in rodents based on publication year, continent, WHO region, country and sample size.

Subgroup variable	Prevalence % (95% CI)	Heterogeneity (*Q*)	df (*Q*)	*I* ^2^ (%)	*p* value
Publication year					
2013–2016	16.3 (10.6–24.3)	8.5	7	17.4	0.05
2017–2020	20.6 (13.7–29.7)	192.9	26	86.5	0.05
2021–2024	13.8 (11.1–19.9)	541.6	42	92.2	0.05
Continent					
Africa	25 (3.4–76.2)	0	0	0	0.05
Asia	12.9 (9.8–17)	647.1	54	91.6	0.05
Europe	35.2 (21.3–52.1)	37.7	14	62.9	0.05
North America	22.2 (8.7–46.1)	10.1	3	70.3	0.05
South America	30.1 (7.6–69.1)	9.1	2	77.9	0.05
WHO region					
AMR	25.6 (13–44)	19.5	6	69.2	0.05
EMR	17.7 (9.1–31.5)	77.5	9	88.4	0.05
EUR	35.2 (21.3–52.1)	37.7	14	62.9	0.05
SEAR	20.6 (8.7–41.6)	13.2	6	54.5	0.05
WPR	11.4 (8.1–15.7)	551	38	93.1	0.05
Country					
Belgium	40 (10–80)	0	0	0	0.05
Brazil	29.5 (2.4–87.9)	9	1	88.9	0.05
China	11.1 (8.1–15)	417	39	90.6	0.05
Colombia	30 (10–62.4)	0	0	0	0.05
France	36 (15.3–63.7)	3	4	0	0.05
Indonesia	13 (7.1–22.5)	0	0	0	0.05
Iran	15.3 (6.4–32.3)	48.7	5	89.7	0.05
Japan	44 (39.3–48.7)	0	0	0	0.05
Libya	25 (3.4–76.2)	0	0	0	0.05
Mexico	22.2 (8.7–46.1)	10.1	3	70.3	0.05
Poland	7.1 (1–37)	0	1	0	0.05
Thailand	23.7 (8.4–51.5)	9.2	5	45.8	0.05
Turkey	61.1 (47.6–73.1)	0	0	0	0.05
The UAE	50 (16.8–83.2)	0	0	0	0.05
The United Kingdom	36.9 (13.2–69.2)	21	5	76.2	0.05
Sample size					
≤100	19.6 (15–25.2)	301.5	59	80.4	0.05
>100	9.9 (6.1–15.7)	426.9	17	96	0.05

### 3.8 Sensitivity Analysis

The sensitivity analysis indicated that excluding certain datasets on *Blastocystis* sp. in rodents did not significantly affect the overall frequency (Figure S6).

### 3.9 Publication Bias

**FIGURE 5:**
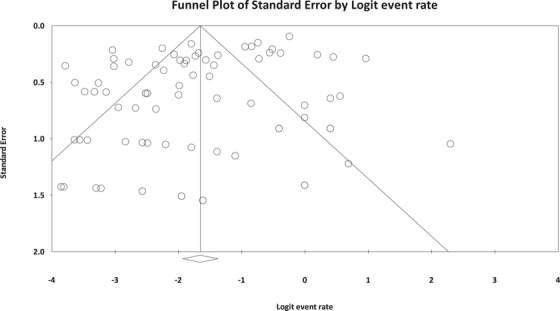
Publication bias in the present study.

The current systematic review and meta‐analysis revealed a notable publication bias (Egger's regression: intercept = −1.591, 95% lower limit = −2.7, 95% upper limit = −0.47, *t* value = 2.83, *p* = 0.005) (Figure 5).

## 4 Discussion

Systematic reviews and meta‐analyses have determined the global prevalence of *Blastocystis* sp. in various animals: cattle (Shams et al. 2021), sheep, goats (Shams, Asghari, et al. 2022), dogs, cats (Shams, Shamsi, et al. 2022) and pigs (Asghari, Sadrebazzaz, et al. 2021), as well as in specific human groups, including haemodialysis, cancer, HIV/AIDS and transplant recipients (Asghari, Sadeghipour, et al. 2021). This study revealed a global prevalence of *Blastocystis* sp. in rodent populations at 16% (95% CI: 12.6%–20.2%), higher than in dogs, cats and immunocompromised individuals, but lower than in cattle, sheep, goats and pigs. Global prevalence refers to the prevalence rate derived from available data, which indicates that differences across animal populations may be influenced by sample size, number of studies, diagnostic methods, animal types, housing and feeding conditions, and geographical location.

Voles and squirrels showed the highest *Blastocystis* sp. infection rates among rodents, whereas civets and porcupines had the lowest, despite variations in dataset numbers and sample sizes across species. This pattern suggests a potential ecological or behavioural influence on the prevalence of *Blastocystis* sp. across different rodent species. For instance, the dietary habits and habitat preferences of voles and squirrels may facilitate more frequent interactions with the pathogen, possibly through higher exposure to contaminated environments or food sources. In contrast, the lower infection rates in civets and porcupines could indicate either a natural resistance to the pathogen or different ecological niches that limit exposure. Furthermore, it is essential to consider the role of host genetics and immune response, which may vary significantly among species. Genetic factors could contribute to varying susceptibilities, influencing how different rodent populations respond to environmental pathogens like *Blastocystis* sp. In addition, the variations in dataset sizes and methodologies used in sampling could introduce biases that affect the accuracy of infection rate assessments. Future studies should aim for standardized sampling techniques across various species to obtain a more comprehensive understanding of the dynamics of *Blastocystis* sp. infections in wildlife.

High genetic diversity in the genus *Blastocystis* sp., as indicated by the small subunit of the ribosomal RNA gene (SSU rRNA), has resulted in its classification into various STs (Stensvold, Alfellani, and Clark 2012). So far, around 44 STs of *Blastocystis* sp. have been identified, with 16 being zoonotic (ST1‐ST10, ST12, ST14, ST16, ST23, ST35 and ST41) and capable of transmission between humans and animals (Liu et al. 2024; Santin et al. 2024). Of note, the genetic diversity of *Blastocystis* sp. extends beyond these 44 STs, and forthcoming studies are expected to uncover additional animal and zoonotic STs. The current findings indicate that among the 12 STs (ST1–ST8, ST10, ST13, ST15, ST17) isolated from rodents, 9 STs (ST1–ST8 and ST10) possess zoonotic properties, highlighting the crucial role of these animals in transmitting *Blastocystis* sp. zoonotic infections to vulnerable human populations and vice versa. Of note, to unequivocally demonstrate the zoonotic transmission of *Blastocystis* sp. between humans and animals, two conditions must be met: Both must coexist in the same spatiotemporal context, and they must possess the same species and genotype. Transmission of *Blastocystis* sp. infection from wild rodents outside human habitats can be relatively low. In contrast, commensal rodents near human dwellings can transmit various microorganisms, including *Blastocystis* sp., to humans and pets, potentially leading to mild to severe health complications (Himsworth et al. 2013; Dahmana et al. 2020; Islam et al. 2021).

The rodent‐specific ST (ST4) was the most common globally among rodents, followed by ST5, ST1, ST3, ST2, ST13, ST17, ST6, ST7, ST8, ST10 and ST15. Several of these STs (ST1–ST8 and ST10) have zoonotic potential, having been isolated from humans and other animals as well. This underscores the need to monitor these STs in both wildlife and domestic populations, as their presence in various hosts can reveal insights into their evolutionary dynamics and potential public health impacts. The country‐based distribution of *Blastocystis* sp. STs in rodents showed that the highest sample numbers came from China, Japan, Iran, the United Kingdom and Mexico, respectively. China and the United Kingdom displayed the most ST diversity, with 10 (ST1–ST7, ST10, ST13, ST17) and 7 (ST1–ST5, ST10, ST15) distinct STs, respectively. Notably, ST6, ST7 and ST13 were exclusive to China, whereas ST15 was found only in the United Kingdom. The distribution patterns of *Blastocystis* sp. STs across various regions suggest that ecological and environmental factors significantly influence these communities. The exclusive presence of ST6, ST7 and ST13 in China, along with ST15 in the United Kingdom, raises important questions about the evolutionary pathways and host interactions of *Blastocystis* sp. in different rodent species. Ongoing research into the genetic characteristics and environmental links of these STs is vital for understanding their epidemiology and potential zoonotic transmission risks. Analysing these biogeographical patterns underscores the value of localized studies in elucidating the ecological relationships between *Blastocystis* sp. and their rodent hosts.

Species‐based analysis revealed that most rodent samples came from rats, squirrels, mice, coypus, civets, porcupines and voles. Squirrels, rats, mice and voles exhibited the highest ST diversity of *Blastocystis* sp., with 8 (ST1‐ST6, ST13, ST17), 7 (ST1‐ST5, ST7, ST17), 5 (ST1, ST3–ST5, ST10) and 5 (ST1, ST4, ST5, ST10, ST15) distinct STs, respectively. Notably, ST6 and ST13 were found only in squirrels, ST7 exclusively in rats and ST15 solely in voles. The analysis indicated a significant variation in the prevalence of the distinct STs among the different rodent species, suggesting potential host‐specificity. For instance, although both squirrels and rats exhibited a rich diversity of STs, the presence of STs such as ST6 and ST13 in squirrels might indicate a particular ecological niche or dietary preference that supports these STs of *Blastocystis* sp. Conversely, the exclusive occurrence of ST7 in rats could suggest a specialized adaptation of this ST to the physiological or environmental conditions unique to the rat habitats. On the other hand, contamination of rodents with human and animal STs in various environments can lead to ST diversity among rodent species. Thus, isolating specific STs from a particular rodent species does not necessarily indicate that subtype's exclusivity to that rodent. However, further investigations into the geographical distribution of these rodent species may shed light on the dynamics of *Blastocystis* sp. transmission. Understanding the interactions between rodent hosts and their associated environmental factors could reveal critical insights into the epidemiology of *Blastocystis* sp.

Most *Blastocystis* sp. STs in rodents were reported in Asia, with ST6, ST7 and ST13 isolated exclusively from there. ST15 was found only in Europe, ST17 was unique to Africa, and both ST4 and ST17 were reported in North America, whereas ST1–ST3 and ST8 were from South America. The distribution of *Blastocystis* sp. STs highlights the intricate interplay between host species and environmental factors. In addition to the geographic isolation of specific STs, studies indicate that the prevalence of certain STs may be influenced by rodent population densities and local ecological conditions. Moreover, the genetic diversity observed within these STs offers insights into their evolutionary pathways and adaptation mechanisms. Overall, the limited studies and narrow geographical focus hinder a thorough investigation of *Blastocystis* sp. STs in specific regions and rodent species, a discussion that will improve as research in this area advances.

The sensitivity analysis showed that there was no outlier data among the 34 studies/78 datasets entered, and no statistically significant change was reported in the prevalence of *Blastocystis* sp. in rodents by removing individual datasets. The subgroup analysis revealed no direct association between publication year and the prevalence of *Blastocystis* sp. in rodents, with the highest prevalence recorded at 20.6% (95% CI: 13.7%–29.7%) between 2017 and 2020. Rodents in Europe and the EUR WHO region exhibited a higher prevalence of *Blastocystis* sp. infection, based on 14 analysed datasets. Among the countries studied, Turkey (61.1%; 95% CI: 47.6%–73.1%), the UAE (50%; 95% CI: 16.8%–83.2%), Japan (44%; 95% CI: 39.3%–48.7%) and Belgium (40%; 95% CI: 10%–80%) showed the highest infection rates compared to the other 11 evaluated countries. A global study found a direct link between larger sample sizes and reduced prevalence of *Blastocystis* sp. in rodents, suggesting that epidemiological studies need sufficient sample sizes to accurately reflect disease or infection prevalence. Overall, due to challenges in identifying wild rodents, mixing of nearby rodent groups and solitary animals, the interpretation of sample numbers and *Blastocystis* sp. prevalence in rodents may involve various errors.

Of note, it is important to emphasize the importance of cats and dogs as key intermediate hosts in the transmission of parasitic infections, such as *Blastocystis* sp., between rodents and humans. These animals serve as significant vectors in the zoonotic transmission of various pathogens, acting as reservoirs that facilitate the lifecycle of parasites. Their interactions with both wild and domestic rodent populations create a dynamic ecological relationship that heightens the risk of infection in humans. As these pets come into contact with contaminated environments, such as parks, yards, or homes where rodents may reside, they inadvertently carry and spread infectious agents like *Blastocystis* sp. (Morelli et al. 2021; Macpherson et al. 2022). Moreover, new pets like ferrets, weasels and polecats consume small mammals such as rabbits, mice, rats and other rodents, serving as reservoirs for parasitic infections like *Blastocystis* sp., which facilitate the human infections and lifecycle of parasites. The environmental contamination from their faeces can spread pathogens, creating a cycle that may inadvertently infect humans (Powers 2009; Vinke and Schoemaker 2012). Therefore, pet owners must adhere to strict hygiene practices, including regular cage cleaning and proper waste disposal.

To prevent the transmission of parasitic infections such as *Blastocystis* sp. from rodents to humans and domestic animals, the following measures can be taken: (1) Utilize integrated pest management (IPM) strategies to reduce rodent populations through traps and environmental management to eliminate food and shelter sources. (2) Maintenance of cleanliness in and around homes, farms and food storage areas to deter rodent infestations by properly disposing of garbage and securely storing food. (3) Encouraging regular handwashing, especially after handling animals, cleaning up rodent droppings, or working in areas where rodents may be present. (4) Educating communities about the risks of rodent‐borne parasites and the importance of control measures. (5) Monitoring rodent populations and potential parasitic infections within local wildlife and domestic animals to quickly identify and respond to outbreaks. (6) Ensuring regular veterinary check‐ups for pets and livestock to detect and treat any parasitic infections early. (7) Environmental modification to reduce rodent access to buildings by sealing entry points and removing debris or clutter (Thompson, Kutz, and Smith 2009; Esch and Petersen 2013; Mackenstedt, Jenkins, and Romig 2015; Mustapha et al. 2019). Implementing these measures can significantly reduce the risk of parasite transmission from rodents to humans and domestic animals.

The current study found significant publication bias among the included studies, likely due to factors such as lack of studies in various geographical areas, the existence of a large number of studies on certain species of rodents and so forth (Kicinski 2013). This study, like many reviews, has limitations, primarily due to analyses based on single studies or datasets and limited geographical diversity. Therefore, the present results should be interpreted with care and caution.

## 5 Conclusion

This systematic review and meta‐analysis revealed a global *Blastocystis* sp. infection rate of 16% in rodent populations, underscoring the influence of ecological and behavioural factors, particularly among voles and squirrels, on pathogen exposure. However, the number of included studies and examined samples of different species in this interpretation should not be ignored. The identification of 12 STs, 9 of which have zoonotic potential, highlights rodents as significant reservoirs for zoonotic transmission, especially in urban settings. The study calls attention to the geographical diversity of these STs, particularly in China and the United Kingdom, emphasizing the role of local conditions in *Blastocystis* sp. epidemiology. Moreover, the need for standardized research methods is critical due to identified publication bias, which hampers accurate prevalence estimates. Ongoing research should expand geographic focus and explore rodent‐environment interactions to clarify transmission pathways. Overall, our findings stress the necessity of monitoring *Blastocystis* sp. in wildlife and domestic populations, alongside effective public health measures to mitigate zoonotic risks and protect vulnerable human communities.

## Author Contributions

Planned and designed the study: Ali Asghari and Amir Farzam. Involved in the methodology: Ali Asghari, Amin Karampour, Seyedeh‐Sara Nazem‐Sadati and Parisa Sadat‐Madani. Conducted the statistical analysis: Ali Asghari. Wrote the manuscript and revised it: Ali Asghari and Amir Farzam. All authors have read and approved the final manuscript.

## Conflicts of Interest

The authors declare no conflicts of interest.

## Supporting information



Figure S1

Figure S2

Figure S3

Figure S4

Figure S5

Figure S6

Figure S7

## Data Availability

The datasets used and/or analysed during the current study are available in the online version.
