# Supplementary material for: Global Prevalence and Subtype Distribution of Blastocystis sp. in Rodent Populations: A Systematic Review and Meta‐Analysis
Source: Vet Med Sci. 2024 Dec 30;11(1):e70178. doi: 10.1002/vms3.70178 (PMC11683779; doi:10.1002/vms3.70178)
Supplement: Supplementary file 3 — Figure S3 [file VMS3-11-e70178-s006.docx]

**Supplementary Fig. 3.** The global prevalence of *Blastocystis* sp. in rodents based on country.
