# Supplementary material for: Global Prevalence and Subtype Distribution of Blastocystis sp. in Rodent Populations: A Systematic Review and Meta‐Analysis
Source: Vet Med Sci. 2024 Dec 30;11(1):e70178. doi: 10.1002/vms3.70178 (PMC11683779; doi:10.1002/vms3.70178)
Supplement: Supplementary file 7 — Figure S7 [file VMS3-11-e70178-s002.docx]

**Supplementary Table 1**

**JBI critical appraisal checklist applied for included studies**

| Author Name/Year | Sample was representative? | Participants appropriately recruited? | Sample size was adequate? | Study subjects and the setting described? | Data analysis conducted | Objective, standard criteria, reliably used? | Appropriate statistical analysis used | Confounding factors/ subgroups/ differences identified and accounted? | Subpopulations identified using objective criteria | Overall quality |
| --- | --- | --- | --- | --- | --- | --- | --- | --- | --- | --- |
| Alfellani, 2013 | Yes | Yes | Yes | Yes | Yes | Yes | No | No | No | 4/9 |
| Ramirez, 2014 | Yes | No | Yes | Yes | No | Yes | No | No | No | 6/9 |
| Seifollahi, 2016 | Yes | No | Yes | No | Yes | No | Yes | No | No | 4/9 |
| Yoshikawa, 2016 | Yes | Yes | No | Yes | No | Yes | No | Yes | No | 5/9 |
| Cian, 2017 | Yes | Yes | Yes | Yes | Yes | No | Yes | Yes | No | 7/9 |
| Betts, 2018 | Yes | Yes | Yes | Yes | Yes | No | Yes | No | No | 6/9 |
| Mohaghegh, 2018 | Yes | Yes | Yes | Yes | Yes | No | Yes | Yes | Yes | 8/9 |
| Wang, 2018 | Yes | Yes | Yes | Yes | Yes | Yes | Yes | Yes | No | 8/9 |
| Xiao, 2019 | Yes | Yes | Yes | Yes | Yes | No | Yes | No | Yes | 7/9 |
| AbuOdeh, 2019 | Yes | Yes | Yes | Yes | Yes | No | Yes | Yes | Yes | 8/9 |
| Valenca-Barbosa, 2019 | Yes | No | Yes | Yes | Yes | Yes | Yes | No | Yes | 7/9 |
| Oliveira-Arbex, 2020 | Yes | Yes | Yes | Yes | Yes | Yes | No | No | No | 4/9 |
| Chai, 2020 | Yes | No | Yes | Yes | No | Yes | No | No | No | 6/9 |
| Li, 2020a | Yes | No | Yes | No | Yes | No | Yes | No | No | 4/9 |
| Li, 2020b | Yes | Yes | No | Yes | No | Yes | No | Yes | No | 5/9 |
| Martinez-Hernandez, 2020 | Yes | Yes | Yes | Yes | Yes | No | Yes | Yes | No | 7/9 |
| Betts, 2020 | Yes | Yes | Yes | Yes | Yes | No | Yes | No | No | 6/9 |
| Mohammadpour, 2020 | Yes | Yes | Yes | Yes | Yes | No | Yes | Yes | Yes | 8/9 |
| Rudzinska, 2021 | Yes | Yes | Yes | Yes | Yes | Yes | No | No | No | 4/9 |
| Liu, 2021 | Yes | No | Yes | Yes | No | Yes | No | No | No | 6/9 |
| Masuda, 2021 | Yes | No | Yes | No | Yes | No | Yes | No | No | 4/9 |
| Chen, 2021 | Yes | Yes | No | Yes | No | Yes | No | Yes | No | 5/9 |
| Song, 2021 | Yes | Yes | Yes | Yes | Yes | Yes | No | No | No | 4/9 |
| Malatyali, 2021 | Yes | No | Yes | Yes | No | Yes | No | No | No | 6/9 |
| Deng, 2021 | Yes | No | Yes | No | Yes | No | Yes | No | No | 4/9 |
| Liu, 2022 | Yes | Yes | No | Yes | No | Yes | No | Yes | No | 5/9 |
| Tantrawatpan, 2023 | Yes | Yes | Yes | Yes | Yes | No | Yes | Yes | No | 7/9 |
| Zhao, 2023 | Yes | Yes | Yes | Yes | Yes | No | Yes | No | No | 6/9 |
| Liu, 2024 | Yes | Yes | Yes | Yes | Yes | No | Yes | Yes | Yes | 8/9 |
| Shan, 2024 | Yes | Yes | Yes | Yes | Yes | Yes | Yes | Yes | No | 8/9 |
| Martinez-Hernandez, 2024 | Yes | Yes | Yes | Yes | Yes | Yes | No | No | No | 4/9 |
| Bastaminejad, 2024 | Yes | No | Yes | Yes | No | Yes | No | No | No | 6/9 |
| Wang, 2024 | Yes | No | Yes | No | Yes | No | Yes | No | No | 4/9 |
| Gao, 2024 | Yes | Yes | No | Yes | No | Yes | No | Yes | No | 5/9 |
